# Involvement of ciliary neurotrophic factor in early diabetic retinal neuropathy in streptozotocin-induced diabetic rats

**DOI:** 10.1038/s41433-018-0110-7

**Published:** 2018-05-23

**Authors:** Mingming Ma, Yupeng Xu, Shuyu Xiong, Jian Zhang, Qing Gu, Bilian Ke, Xun Xu

**Affiliations:** 1 Shanghai Key Laboratory of Ocular Fundus Diseases, Shanghai, China; 2 Department of Ophthalmology, Shanghai General Hospital, Shanghai, China; 3 Shanghai Engineering Center for Visual Science and Photomedicine, Shanghai, China

## Abstract

**Objective:**

Ciliary neurotrophic factor (CNTF) has been evaluated as a candidate therapeutic agent for diabetes and its neural complications. However, its role in diabetic retinopathy has not been fully elucidated.

**Methods:**

This is a randomized unblinded animal experiment. Wistar rats with streptozocin (STZ)-induced diabetes were regularly injected with CNTF or vehicle control in their vitreous bodies beginning at 2 weeks after STZ injection. A total of five injections were used. In diabetic rats, the levels of CNTF and neurotrophin-3 (NT-3) were evaluated by enzyme-linked immunosorbent assays (ELISA) and real-time PCR. The abundance of tyrosine hydroxylase (TH) and *β*-III tubulin was detected by western blot. Transferase-mediated dUTP nick-end labeling staining (TUNEL) was used to detect cell apoptosis in the retinal tissue. The activation of caspase-3 was also measured.

**Results:**

The protein and mRNA levels of CNTF in diabetic rat retinas were reduced compared to control rats. In addition, retinal ganglion cells (RGCs) and dopaminergic amacrine cells appeared to undergo degeneration in diabetic rat retinas, as revealed by transferase-mediated dUTP nick-end labeling staining (TUNEL). Tyrosine hydroxylase (TH) and *β*-III tubulin protein levels also decreased significantly. Intraocular administration of CNTF rescued RGCs and dopaminergic amacrine cells from neurodegeneration and counteracted the downregulation of *β*-III tubulin and TH expression, thus demonstrating its therapeutic potential.

**Conclusion:**

Our study suggests that early diabetic retinal neuropathy involves the reduced expression of CNTF and can be ameliorated by an exogenous supply of this neurotrophin.

## Introduction

Diabetic retinopathy (DR) is the leading cause of visual impairment and preventable blindness throughout the world [1, 2], representing a significant socioeconomic cost to healthcare systems [3–5]. DR is also the most common complication of diabetes and afflicts roughly one-third of the diabetic population. Given that the incidence of diabetes is expected to increase from 366 million in 2011 to 552 million in 2030, DR will likely become an even more prevalent and serious problem in the future [6]. The substantial worldwide public health burden of diabetes and its resulting complications (e.g., DR) underscores the immediate need to identify new approaches to prevent such complications, which go beyond the current standards of diabetic care.

Current treatments for DR include laser photocoagulation and the administration of anti-vascular endothelial growth factor (VEGF). Laser photocoagulation is applicable only at advanced stages of disease and is associated with significant adverse effects, such as moderate vision loss, reduced color vision, reduced contrast sensitivity [7], infection, and glaucoma [8]. Vitreoretinal surgery is another option, but it is an expensive treatment that can only be performed by experienced vireoretinal surgeons and is therefore typically reserved for cases where blindness occurs due to proliferative DR (PDR) [9]. Treatment in the early stages of DR is critical in slowing or preventing disease progression (leading to blindness). However, no drugs are currently available that effectively prevent the incidence or progression of DR in these critical early stages. Therefore, new pharmacological treatments are desperately needed.

DR has long been considered a microvascular disease, and blood–retinal barrier (BRB) breakdown is a hallmark of this disease. However, it is now wildly recognized as a neuro-vascular disease. In streptozotocin (STZ)-induced diabetic rats, vascular changes are observed, including microaneurysms, decreases in pericyte numbers, increased vascular permeability, BRB breakdown, and early growth factor changes that are characteristic of background/non-proliferative DR. More neuronal cells undergo apoptosis in the retinas of early stage diabetic versus control rats [10, 11]. Others have also observed losses of the axonal fiber in diabetic rat retinas [12, 13].

Numerous early retinal function tests in DR patients suggest that neurons are vulnerable to damage shortly after the onset of diabetes, long before any sign of vascular damage is apparent [10, 14]. Multifocal electroretinography (ERG), flash ERG, contrast sensitivity, color vision, and short-wavelength-automated perimetry examinations have detected functional deficits in the neuronal component of diabetic retinas during the early stages of the disease [15, 16]. Thus, neuron dysfunction or degeneration and vascular alterations may be characteristics of early diabetic retinopathy.

CNTF is a member of the IL6 family of cytokines, which includes leukemia inhibitory factor, CNTF, IL11, and IL6 itself [17]. CNTF is distributed throughout the central and peripheral nervous system in glial cells and neurons [18, 19] and supports the differentiation and/or survival of a variety of neurons during development [20]. The receptor for CNTF includes a glycosylphosphatidylinositol-linked α component, CNTFRα, which is present in nerves and muscle [21, 22] and a transmembrane β component (the LIFRβ/GP130 heterodimer) that is localized to nerves and macrophages [23, 24]. CNTF can enhance photoreceptor survival, as demonstrated in multiple animal models of retinal degeneration, ranging from zebrafish to canine models [25]. CNTF is effective in prolonging the survival of retinal ganglion cells [26–28] and promoting axonal growth in optic nerve crush and transaction models [29–31]. In addition, CNTF can rescue retinal degeneration due to various causes, including mutations in genes expressed by photoreceptors or the retinal pigment epithelium (RPE), as well as those induced by strong light or neurotoxins [32, 33]. A secreted form of human CNTF that is delivered from an encapsulated cell device has been tested in clinical trials, based on its significant and broad neuroprotective effects in damaged retinas. This therapy has been approved to treat retinitis pigmentosa (RP) and geographic atrophy (GA), which represent a subset of age-related macular degeneration (AMD) conditions [34–36].

## Materials and methods

### Reagents

TriZol was purchased from Invitrogen (Grand Island, NY). Reverse transcription and RT-PCR systems were purchased from Roche (Mannheim, Germany) and Takara (Japan), respectively. All other reagents were obtained from Sigma (St. Louis, MO). For western blot analyses, a rabbit polyclonal antibody against tyrosine hydroxylase (TH) was purchased from Cell Signaling Technology (Beverly, MA), a rabbit monoclonal antibody against *β*-III tubulin antibody was obtained from Abcam (MA, USA), and an antibody to rat GAPDH was obtained from Epitomics (CA, USA). NT-3 ELISA kits were purchased from R&D Systems (MN, USA), and CNTF ELISA kits were purchased from Sigma (St. Louis, MO). The secondary antibody was purchased from Santa Cruz Biotechnology.

### Animals

Adult male Wistar rats (250–300 g) were obtained from the Shanghai Laboratory Animal Center (Shanghai, China). All animals were housed in a 12-h light-dark cycle. Animals were randomly divided into a diabetic and non-diabetic group according to the method of random number table. Diabetes was induced by daily intraperitoneal injections of streptozotocin (STZ; Sigma, St. Louis, MO) at 65 mg/kg body weight for 3 days. Age-matched non-diabetic animals were injected with an equal volume of citrate buffer. Body weight and blood glucose concentrations were measured before injection and weekly thereafter. Diabetes was confirmed by assaying the glucose concentration in blood collected from the tail vein using a commercially available diagnostic kit (Human, Wiesbaden, Germany). Rats with glucose levels >300 mg/dl were classified as diabetic 5 days after first injection of STZ. At the study conclusion (4 weeks post-injection), rats were anaesthetitised with chloral hydrate and decapitated to facilitate retinal dissection. Eyes with dense cataracts, vitreous hemorrhages, retinal detachment, or death were excluded.

The experimental protocols used in this study followed guidelines established by the ARVO Statement for the Use of Animals in Ophthalmic and Vision Research and were approved by the Ethics Committee of Shanghai First People’s Hospital, Shanghai Jiaotong University, Shanghai, China (Permit Number: 2009-0086).

### Intraocular administration of CNTF

Multiple intraocular injections, for a total of five doses, were given every 3 days, beginning 2 weeks after the intraperitoneal injection of STZ or citrate buffer. Levofloxacin hydrochloride eye drops 0.5% (Santen Pharmaceutical, Noto, Japan) were applied to the ocular surface before injection. A drop of 0.4% oxybuprocaine hydrochloride (Santen Pharmaceutical, Osaka, Japan) was used for additional topical anesthesia. CNTF (5 µg) in 5 µl balanced salt solution (BSS) (Alcon, Fort Worth, TX) was injected into the vitreous body of one eye chosen at random according to a previous report [37]. An equal volume of vehicle was injected into the other eye as a control. Retinas were dissected 2 days after the last intraocular injection. Animals with lens damage, vitreous hemorrhage, or retinal detachment were excluded from the analyses.

### Enzyme-linked immunosorbent assay

Protein levels of CNTF and neurotrophin-3 (NT-3) were quantified by enzyme-linked immunosorbent assays (ELISAs), according to the manufacturer’s instructions. Simply, the retinas (*n* = 4 rats in each group) of diabetic and non-diabetic rats were isolated and placed into 200 µl lysis buffer (0.05 M Tris-HCl, pH 7.4, 0.15 M NaCl, 0.25% deoxycholic acid, 1% NP-40, 1 mM EDTA) supplemented with protease inhibitor (Roche, Mannheim, Germany), which was then sonicated. The lysate was centrifuged at 25,436 × *g* for 20 min at 4 °C. The protein levels of CNTF and NT-3 were measured according to the manufacturer’s protocols. The average value of the sample was normalized against the total protein concentration.

### RNA isolation and RT-PCR

mRNA levels of CNTF and NT-3 in the retinal tissue were quantified by real-time PCR. Total RNA was extracted from retinas as previously described [38]. The quality of the mRNA was evaluated by the ratio of 28S ribosomal RNA (rRNA) to 18S rRNA; samples (*n* = 4 rats in each group) with ratios >1.6 were used for real-time PCR (*n* = 3 per group) [39]. Total RNA (50 ng) was amplified using an RT-PCR kit (Takara, Japan). The sequences of the primers were as follows: CNTF, 5′-CGACTCCAAGAGAACCTCCA-3′ and 5′- CCTTCAGTTGGGGTGAAATG-3′ [40], NT-3, 5′-GATCCAGGCGGATATCTTGA-3′ and 5′-AGCGTCTCTGTTGCCGTAGT-3′ [41], β-actin sense 5′-CACTGCCGCATCCTCTTCCTC-3′ and antisense 5′-TGCTGTCGCCTTCACCGTTCC-3′. Each experiment was performed a total of three times. Primers for β-actin were designed using the Primer-BLAST primer design tool at NCBI (http://www.ncbi.nlm.nih.gov/tools/primer-blast/index.cgi?LINK_LOC = BlastHome). β-actin served as the internal control. Each PCR reaction contained 0.5 µM primers, 200 µM dNTPs, 1.5 µM MgCl_2_, 1.25 U of Taq polymerase, and 1 µl cDNA. The parameters were set as follows: 37 °C for 1 h, 95 °C for 5 min, and 45 amplification cycles of 95 °C for 5 s and 60 °C for 20 s. Relative mRNA was normalized to β-actin, and fold-changes were calculated using the 2^−ΔΔCT^ method as described previously [42].

### Western immunoblot analyses

To determine the relative TH and *β*-III tubulin protein levels, retinal tissues were lysed in RIPA lysis buffer (0.05 M Tris-HCl, pH 7.4, 0.15 M NaCl, 0.25% deoxycholic acid, 1% NP-40, 1 mM EDTA.). The protein samples (*n* = 4 rats in each group) were subjected to SDS-PAGE followed by western blotting, as previously reported [43]. A rabbit anti-TH polyclonal antibody (1:1000 dilution (Cell Signaling Technology, Beverly, MA, USA) and a rabbit anti-*β*-III tubulin monoclonal antibody (1:3000 dilution, Abcam, Cambridge, MA, USA) were used as the primary antibodies. Peroxidase-conjugated goat anti-rat IgG (1:2000 dilution, Santa Cruz Biotechnology, Inc., Santa Cruz, CA) was used as the second antibody. Protein levels of CNTFRα (the receptor for CNTF) in the retina were determined by western blot using an anti-CNTFRα antibody (1:3000 dilution, Abcam, Cambridge, MA, USA). Anti-GAPDH (1:1000 dilution, Cell Signaling Technology, Beverly, MA, USA) was used as a loading control for western blotting. The intensity of the bands was quantified by densitometry using ImageJ software (NIH, USA).

### Immunohistochemistry and transferase-mediated dUTP nick-end labeling staining

To detect individual apoptotic cells, dual-color staining for transferase-mediated dUTP nick-end labeling staining (TUNEL) and TH protein (Abcam, Shatin, Hong Kong) was carried out using a DeadEnd™ Fluorometric TUNEL System kit (Promega, Madison, WI, USA) according to the manufacturer’s instructions. Cell nuclei were counterstained with DAPI (1 μg/ml; Beyotime Institute of Biotechnology, Jiangsu, China) to visualize the layer structure of the retina. Samples (*n* = 6 animals in each group) were observed under a confocal laser scanning microscope (Zeiss LSM510; Carl Zeiss, Thornwood, NY). The numbers of total and TUNEL-positive nuclei in the ganglion cell layer (GCL) were counted per length of section as previously described [44].

### Measurement of caspase-3 activity

The enzymatic activity of the caspase-3 class of proteases in the retina (*n* = 6 animals in each group) was measured by a caspase-3 colorimetric assay kit (Promega, Madison, WI, USA) per the manufacturer’s instructions. Briefly, the enzymatic reaction for caspase activity was performed by the addition of 50 µl of retina homogenate from a total of 150 µl/retina in a 96-well flat bottom microplate. The cleavage of a caspase-3 colorimetric substrate (DEVD-pNA) was measured at 405 nm using a microplate reader (Auto Bio Labtech Instruments, Co, Ltd, China).

### Data analyses

The sample size was estimated according to the previous study [41]. All values are expressed as mean ± SD. Statistical significance between groups was analyzed either by Welch’s *t* test or one-way ANOVA followed by Fisher’s projected least significant difference (PLSD) multiple comparison tests, as indicated. *P* < 0.05 was considered statistically significant. All statistical routines were used as implemented in SPSS version 17.0 (IBM).

## Results

### Reduced TH and *β*-III tubulin protein levels in diabetic rat retinas

**Fig. 1:**
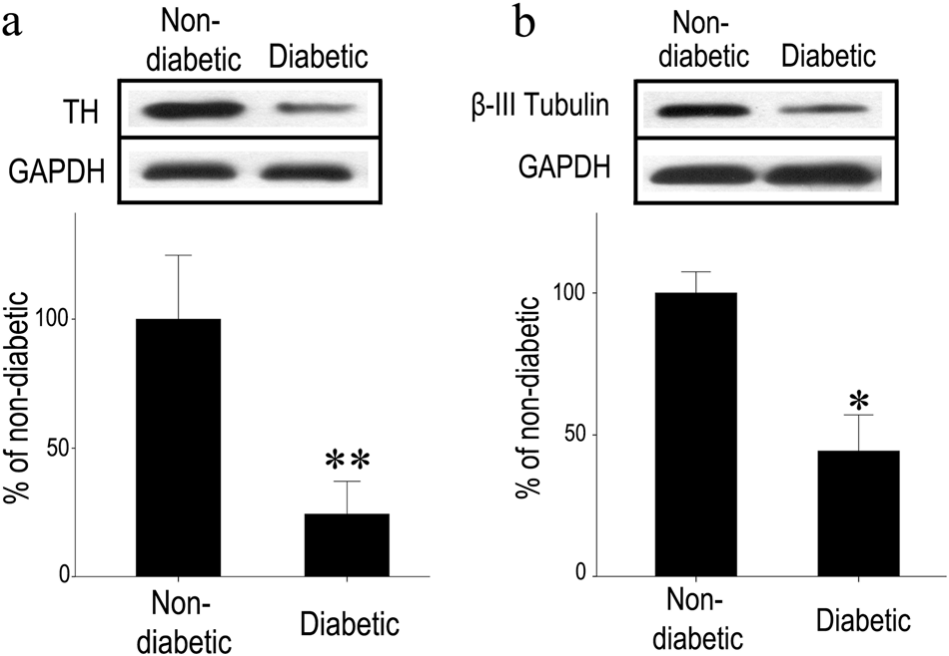
Reduced TH and β-tubulin protein levels in diabetic rat retinas. **a** Western blotting was used for TH, β-tubulin, and GAPDH in the retinas. **b** Denstitometric analyses of TH and β-tubulin protein levels, standardized against GAPDH protein levels in the lane, were performed. TH and β-tubulin levels in diabetic animals were lower than those of non-diabetic animals. Bars represent the mean ± SD values, with the mean values for non-diabetic rat retinas set at 100%. (Welch’s *t* test, *p < 0.5, **p < 0.01)

TH protein levels, which were used as a marker for retinal dopaminergic amacrine cells, were quantified via western blot (Fig. 1). The mean value obtained for non-diabetic rats was set as 100% for each protein species, and TH protein levels were standardized to GAPDH levels. We found that TH protein levels were significantly lower in diabetic than in non-diabetic rats (*p* < 0.01, *n* = 4 rats in group) (Fig. 1). We found that the levels of *β*-III tubulin in diabetic rats were also significantly lower than those of normal rats (*p* < 0.05, *n* = 4 rats in each group) (Fig. 1).

### Reduction in the dopaminergic amacrine cells and TH immunoreactivity in the diabetic rat retina

**Fig. 2:**
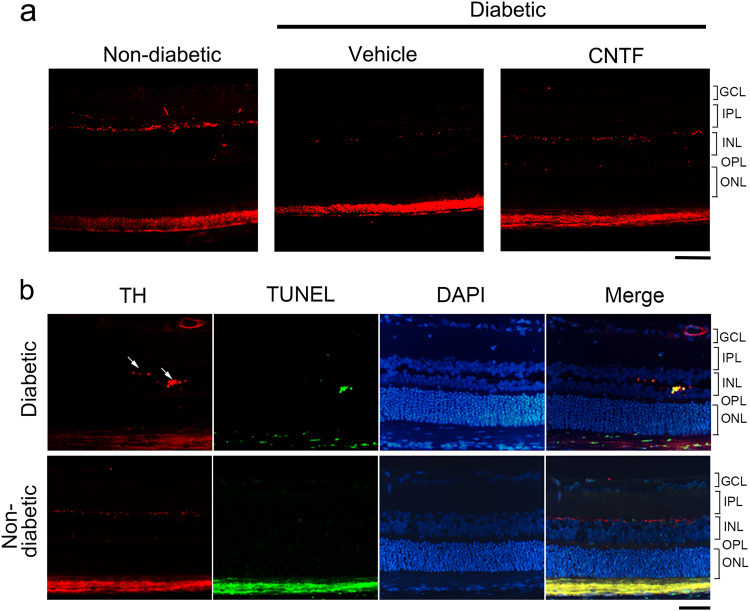
Immunohistochemistry of TH and TUNEL staining on paraffin sections of rat retinas. **a** In the rat retinas, the TH-positive amacrine neurons and fibers were observed in the innermost row of the INL. TH-positive fibers of the IPL could be detected in the non-diabetic rat retina. In the diabetic animals, TH-positive fibers in the IPL cannot be detected, and the fibers in the INL were thinner than in non-diabetic animals. CNTF can reduce the loss of TH-positive cells and fibers in diabetic animal retinas. **b** TUNEL staining was carried out in combination with immunostaining against TH. In the images, the TH-positive neurons (red) showed a TUNEL-positive signal (green), confirming that apoptosis occurs in the dopaminergic amacrine cells of diabetic animals. No apoptosis event was detected in the retinas of non-diabetic animals. Scale bar = 50 μm

n both non-diabetic and diabetic rat retinas, TH-positive amacrine neurons were observed in the inner nuclear layer (INL) (Fig. 2a). TH-positive fibers in the inner plexiform layer (IPL) could be detected in the non-diabetic rat retina (Fig. 2a). In the diabetic animals, TH-positive amacrine neurons in the INL were thinner, and TH-positive fibers in the IPL were hardly visible. When specimens were analyzed by dual-color staining for TUNEL and TH protein, some TH-positive amacrine neurons undergoing apoptosis were detected in diabetic rats (Fig. 2b). TUNEL-positive amacrine neurons were not observed in non-diabetic rats (Fig. 2b).

### Reduction in CNTF, but not NT-3 expression in diabetic rat retinas

**Fig. 3:**
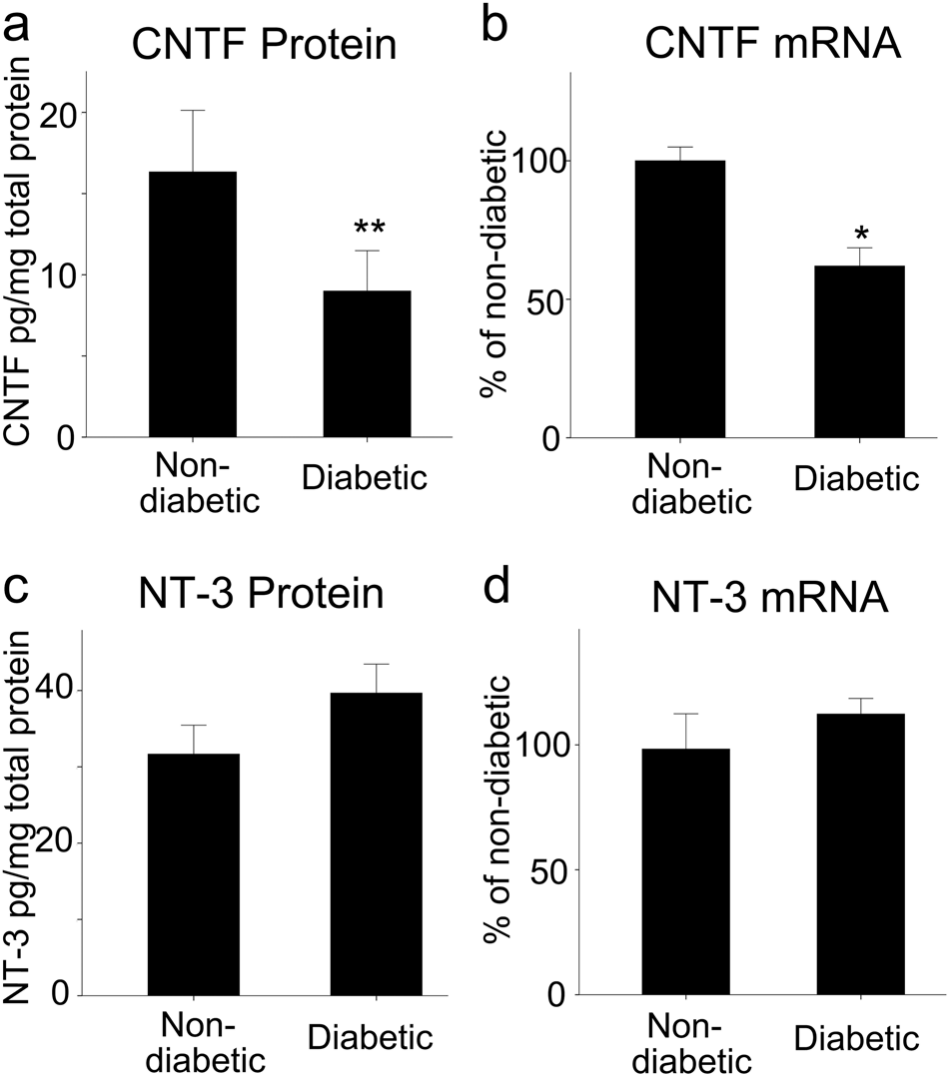
Quantification of CNTF and NT-3 levels in diabetic rat retinas. Protein levels of CNTF (**a**) and NT-3 (**c**) in the retinas of non-diabetic and diabetic rats were quantified by ELISA and were represented as quantities of neurotrophin per total soluble protein. Bars represent mean ± SD. CNTF protein levels in the retinas of diabetic rats were significantly decreased compared with those in non-diabetic rats. In contrast, there were no significant differences in NT-3 protein levels between diabetic rats and non-diabetic rats. mRNA levels of CNTF (**b**) and NT-3 (**d**) were determined by real-time PCR, and their values were standardized to β-actin mRNA levels in the same RNA sample. Bars represent mean ± SD. CNTF mRNA levels in diabetic rats were significantly lower than those of non-diabetic rats, whereas NT-3 mRNA levels were not significantly different between diabetic and non-diabetic animals (Welch’s *t* test, *p *<* 0.5, **p *<* 0.01)

As shown in Fig. 3a, b, CNTF protein levels in the retinas of diabetic rats were significantly decreased compared to control rats (*p* < 0.01, *n* = 4 rats in each group). In contrast, no significant differences in NT-3 protein levels were found between diabetic and non-diabetic rats. Trends for mRNA expression echoed those observed for protein levels; CNTF mRNA levels were significantly lower in diabetic animals (*p* < 0.05), while NT-3 mRNA levels did not differ significantly (Fig. 3c, d).

### Therapeutic effect of CNTF in preventing degeneration of neural cells in diabetic rat retinas

**Fig. 4:**
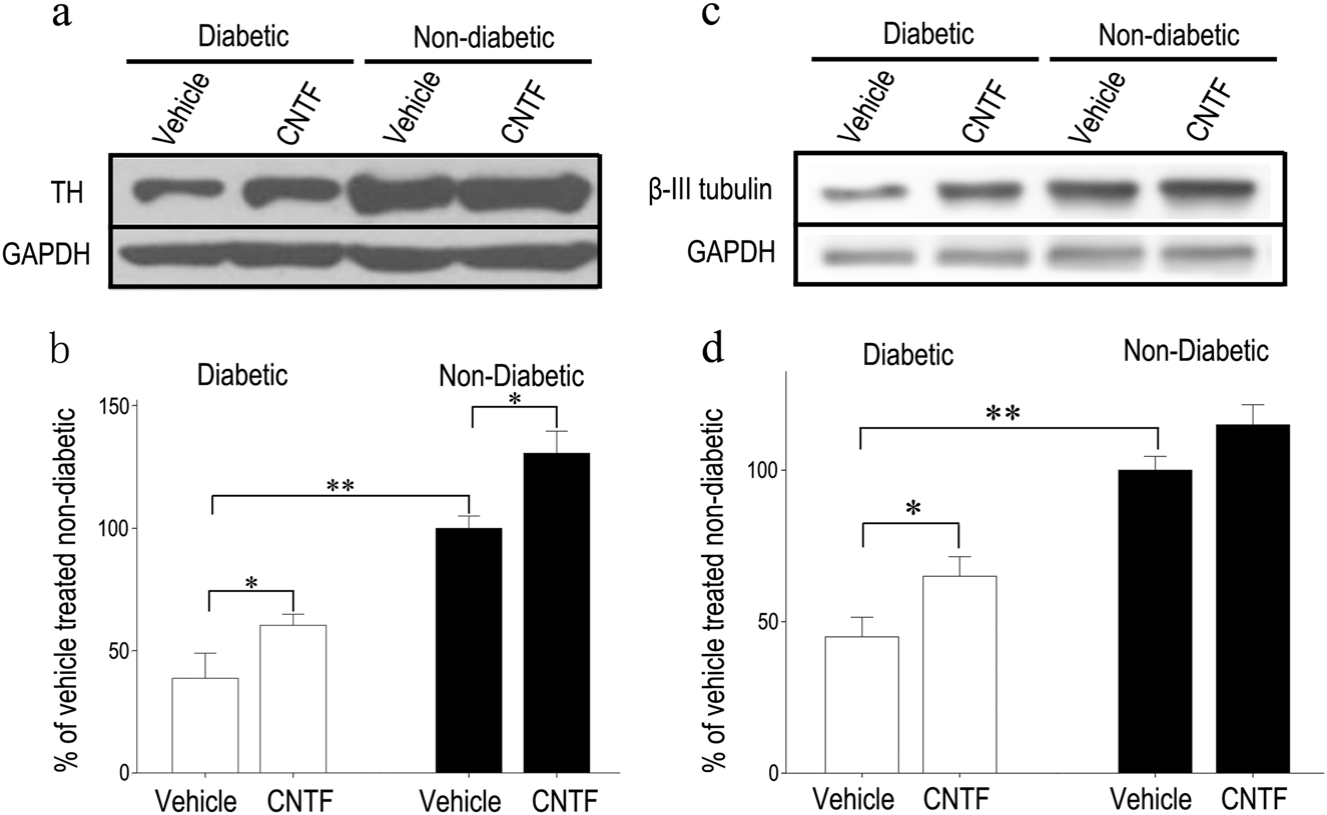
Therapeutic effect of CNTF against degeneration of dopaminergic amacrine cells in diabetic rat retinas demonstrated by western blot. Densitometric analyses of TH (**a**, **b**) and *β*-III tubulin level (**c**, **d**), standardized against GAPDH levels in the same lane, were performed, and the mean value for the vehicle-treated non-diabetic rat retinas was set at 100% (100 ± 8%). Bars represent the means ± SD for each group. One-way ANOVA followed by Fisher’s PLSD multiple comparison tests showed that retinal TH and *β*-III tubulin levels in the CNTF-treated diabetic eyes were higher than those in vehicle-treated diabetic eyes (*p < 0.05; **p < 0.01). The levels of TH in CNTF-treated non-diabetic retinas were higher than those in vehicle-treated non-diabetic retinas (^#^*p* < 0.05)

TH and *β*-III tubulin protein levels were assessed by western blot; vehicle-treated non-diabetic rat retinas was set as 100% (Fig. 4). Retinal TH and *β*-III tubulin levels in the vehicle-treated diabetic eye were markedly reduced (*p* < 0.01) compared to vehicle-treated non-diabetic rat retinas. CNTF treatment also maintained TH and *β*-III tubulin levels in the diabetic rat retinas (*p* < 0.05) compared to vehicle-treated diabetic rat retinas. Furthermore, CNTF application upregulated TH protein levels, even in non-diabetic rats. However, no differences in *β*-III tubulin levels were detected between CNTF-treated and vehicle-treated non-diabetic rat retinas.

**Fig. 5:**
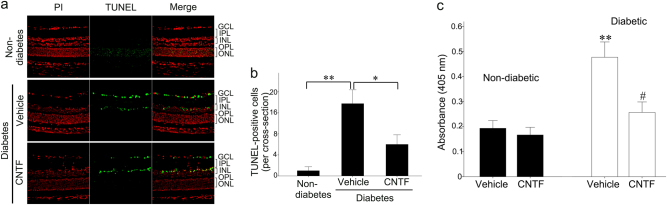
**a**, **b** Diabetes-induced apoptosis was inhibited by CNTF. The cell bodies of the dopaminergic amacrine cells were located in the INL. The green staining cells in the INL of CNTF-treated diabetic retinas were more abundant than in vehicle-treated diabetic retinas. One-way ANOVA followed by Fisher’s PLSD multiple comparison tests showed that the staining in the GCL of diabetic retinas was increased (**p < 0.01), but suppressed significantly by CNTF (*p < 0.05). Values are mean ± SD. Scale bar = 50 μm. **c** CNTF inhibits caspase-3 activity in diabetic rat retinas. Retinal caspase-3 activities in diabetic eyes were significantly higher than those in vehicle-treated non-diabetic eyes. Retinal caspase-3 activities in CNTF-treated diabetic eyes were less than those in vehicle-treated eyes. CNTF application itself only decreased caspase-3 activity in the diabetic rat retina. Data are presented as mean ± SD for each group. One-way ANOVA followed by Fisher’s PLSD multiple comparison tests was used. **p *<* 0.01 in comparison with the vehicle-treated non-diabetic retinas and ^#^*p* *<* 0.05 in comparison with the vehicle-treated diabetic retinas

To investigate the protective effects of CNTF on dopaminergic amacrine cells and GCLs, TUNEL or anti-TH antibody labeling was performed in retinal cross sections after intravitreal CNTF injection. The number of TH-positive cells in the INL of CNTF-treated diabetic retinas was higher than in vehicle-treated diabetic retinas (Fig. 2a). CNTF can reduce the loss of dopaminergic amacrine cells in the early stages of diabetes. TUNEL assays performed on retina sections indicated that the number of TUNEL-positive (green) cells in the GCL of the vehicle-treated diabetic retinas was significantly increased (*p* < 0.001) compared to non-diabetic retinas (Fig. 5a, b). However, CNTF treatment reduced cell death in the GCL of diabetic rat retinas (GCL, *p* < 0.01; INL, *p* < 0.05) compared to vehicle-treated diabetic retinas.

Activation of caspase-3 is a hallmark of apoptotic cell death that precedes changes in nuclear morphology [45]. To confirm the activity of caspase-3 and the effect of CNTF on caspase-3 activity in diabetic or non-diabetic rat retinas, we measured caspase-3 activity in the extracts of diabetic and non-diabetic rats using the chromogenic substrate Ac-DEVD-pNA. Our results show that (Fig. 5c) the intraocular injection procedure itself does not alter retinal caspase-3 activity in normal animals compared with animals not receiving injection (*p* = 0.32). Retinal caspase-3 activity was markedly increased in the vehicle-treated diabetic eye (*p* < 0.01) compared to vehicle-treated non-diabetic eyes. CNTF treatment inhibited the caspase-3 activity in diabetic rat retinas (*p* < 0.05) compared to diabetic retinas not receiving CNTF. However, CNTF application did not affect the activity of caspase-3 in non-diabetic rats.

## Discussion

Researchers examining retinal dopamine content [46] and TH activity [47] have suggested that dysfunction in dopaminergic amacrine cells occurs in the retinas of diabetic rats. In our study, we demonstrated that the degeneration of dopaminergic amacrine cells can occur during the early stages of STZ-induced diabetes. In general, TH and β-III tubulin protein levels in the retina were decreased, reflecting degeneration of dopaminergic amacrine cells and the TH-positive fibers (Figs. 1 and 2).

The degeneration of dopaminergic amacrine cells is a vexing problem in the progression of DR. There are no effective means to treat it, because the mechanisms underlying the degeneration of dopaminergic amacrine cells in diabetic animals are largely unknown. However, several mechanisms have been postulated [48]. The first involves insulin deprivation, which occurs in STZ-induced diabetes. Insulin is an essential factor for the survival of amacrine cells in vitro [49]. Systemically administered insulin can accumulate in the vitreous fluid and augment insulin receptor phosphorylation [50]. A second possible mechanism involves hyperglycemia. High glucose induces cell apoptosis in cultured retinal neural cells [51]. A third possible mechanism involves the generation of reactive oxygen species (ROS) in the retina. Diabetic neurodegenerative changes in the retina can be prevented by suppressing ROS production [52, 53]. The fourth possibility involves the dysfunction of Muller cells in the diabetic retina. Diabetic Muller cells show reduced glutamate-aspartate transporter functions and are compromised in their ability to synthesize glutamine [14, 54]. These dysfunctions result in elevated glutamate [55] levels in diabetic retinas, which may induce excitotoxicity in amacrine cells.

Diabetes-related changes in the expression levels of neurotrophins have been reported in retinas, peripheral nerves, and the brain [56]. However, the CNTF levels in diabetic retinas have not been reported. Here, we show that both the mRNA and protein levels of CNTF are markedly decreased in STZ-induced diabetic rats (Fig. 3). In contrast to the reduction in CNTF levels, NT-3 expression was unaffected.

CNTF has been shown to improve nerve conduction and to repair regeneration deficits in peripheral nerves [57], while potentially preventing the death of retinal amacrine cells [58] in vivo. To confirm the neuropathological implications of reductions in CNTF, we injected exogenous CNTF protein into the vitreous body of diabetic rats. Promisingly, we found that CNTF injection efficiently rescues dopaminergic amacrine cells and RGCs from neurodegeneration and counteracts the downregulation of TH and β-III tubulin levels seen in diabetic retinas. In non-diabetic rats, CNTF can increase protein levels of TH, which indicates that CNTF may improve the activity of the dopaminergic amacrine cells in the retina. However, CNTF has no effect on the protein expression of β-III tubulin in the retina. We propose that CNTF protects neurons from cell death, possibly through CNTFR, which activates downstream Ras/mitogen-activated protein kinase and the Jak/STAT pathway, along with the ERK and the PI3K pathways, which are relevant to neuronal survival. Neurotrophins have biological roles other than aiding neuronal survival; CNTF also supports the differentiation of a variety of neurons during development [20]. Although the neuroprotective effects of CNTF have been reported [59], the precise signaling mechanism(s) by which CNTF protects dopaminergic amacrine cells in diabetic retinas requires further investigation. Previous reports have demonstrated that dopaminergic amacrine cells express CNTFR [60]. Thus, CNTFR is likely to play a role in this process. Caspase-3 is an executioner caspase, relaying downstream death signals and activating other caspase proteins. Another possibility is that CNTF, which has been shown to reduce the apoptosis of pancreatic islet cells and control systemic glucose levels [61], might also act similarly in the retina. In our study, we observed an increase in the activity of caspase-3 in the retina, suggesting that the apparent increase in cell death may be due to caspase activation.

Neurotrophins have been tested as candidate therapeutic agents for diabetic neuropathy. However, neurotrophins do not cross the blood–retina barrier [62], making it difficult to ensure their local supply to the retinal neurons. Although we were able to inject CNTF directly to the vitreous body of the eyeball, frequent injection of neurotrophic factors into the vitreous space of diabetic patients is highly impractical. An intravitreous implant that releases CNTF continuously or the impregnation of CNTF-secreting cells may resolve such problems and could have a better prospect of clinical application.

## Disclaimer

We declare that we have no financial and personal relationships with other people or organizations that can inappropriately influence our work, there is no professional or other personal interest of any nature or kind in any product, service, and/or company that could be construed as influencing the position presented in, or the review of, the manuscript entitled, “Involvement of Ciliary Neurotrophic Factor in Early Diabetic Retinal Neuropathy in Streptozotocin-Induced Diabetic Rats”.

### Summary

#### What was known before

- Neuron dysfunction or degeneration in retina has occurred in early diabetic retinopathy.

#### What this study adds

- CNTF helps to prevent amacrine and neural cell degeneration in the retinas of diabetic rats.
